# Neuroimaging Studies Reveal the Subtle Difference Among Social Network Size Measurements and Shed Light on New Directions

**DOI:** 10.3389/fnins.2018.00461

**Published:** 2018-07-10

**Authors:** Xiaoming Liu, Shen Liu, Ruiqi Huang, Xueli Chen, Yunlu Xie, Ru Ma, Yuzhi Luo, Junjie Bu, Xiaochu Zhang

**Affiliations:** ^1^School of Humanities and Social Science, University of Science and Technology of China, Hefei, China; ^2^School of Foreign Languages, Anhui Jianzhu University, Hefei, China; ^3^CAS Key Laboratory of Brain Function and Disease and School of Life Sciences, University of Science and Technology of China, Hefei, China; ^4^Hefei Medical Research Center on Alcohol Addiction, Anhui Mental Health Center, Hefei, China; ^5^Centers for Biomedical Engineering, University of Science and Technology of China, Hefei, China

**Keywords:** social network size, brain regions, social brain hypothesis, SNI, SNQ

## Abstract

Social network size is a key feature when we explore the constructions of human social networks. Despite the disparate understanding of individuals’ social networks, researchers have reached a consensus that human’s social networks are hierarchically organized with different layers, which represent emotional bonds and interaction frequency. Social brain hypothesis emphasizes the significance of complex and demanding social interaction environments and assumes that the cognitive constraints may have an impact on the social network size. This paper reviews neuroimaging studies on social networks that explored the connection between individuals’ social network size and neural mechanisms and finds that Social Network Index (SNI) and Social Network Questionnaires (SNQs) are the mostly-adopted measurements of one’s social network size. The two assessments have subtle difference in essence as they measure the different sublayers of one’s social network. The former measures the relatively outer sub-layer of one’s stable social relationship, similar to the sympathy group, while the latter assesses the innermost layer—the core of one’s social network, often referred to as support clique. This subtle difference is also corroborated by neuroimaging studies, as SNI-measured social network size is largely correlated with the amygdala, while SNQ-assessed social network size is closely related to both the amygdala and the orbitofrontal cortex. The two brain regions respond to disparate degrees of social closeness, respectively. Finally, it proposes a careful choice among the measurements for specific purposes and some new approaches to assess individuals’ social network size.

## Introduction

Exploration of the features and constructions of human’s social networks has a long history in both the sociological and social anthropological research fields (Lev-Ari, 2018). In contrast to the traditional ecological approaches, the recent attempt to explain the evolution of sociality in primates, known as social brain hypothesis emphasizes the significance of complex social environments in which primates live and assumes that the cognitive constraints may have an impact on social grouping patterns (Liu et al., 2018).

For many species, particularly for primates, living in groups is a major adaptive advantage. But living in a social group also presents its own challenges. To get along while getting ahead, it is necessary to learn who is who, who is friend and who is foe (Bickart et al., 2011). Accordingly, maintaining a stable social group is quite cognitively demanding (Dunbar, 2012). Thus, primate brain evolution was driven by the need to acquire the competence to manage complex social relationships effectively (Dunbar and Shultz, 2007; Liu et al., 2018).

Researches on social networks have concentrated on two major issues. One is to find the limiting size of social networks, while the other is to explain the possible factors that result in the individual difference in social network size. They inevitably raised fundamental questions about the nature of social networks and how they should be defined (Stiller and Dunbar, 2007).

In spite of the disparate definitions of social network among researches, at least one consensus has been reached, that is social networks are hierarchically organized, consisting of different layers, which reflect emotional connection and interaction frequency among individuals (Carmona and Gomila, 2016; Dunbar, 2016; Kardos et al., 2017; Spiegel et al., 2018). The innermost layer (often referred to as support clique) is the core of one’s social network, and is understood as the number of individuals from whom one would seek personal advice or help in case of emotional and financial difficulties (Parkinson et al., 2018). Support cliques are embedded in a larger network that is often discerned as the sympathy group which is a set of individuals one contacts at least once a month and has special ties to. The above-mentioned different levels of social networks constitute an individual’s stable social relationships maintained over a period of time (Stiller and Dunbar, 2007). The outer layers are rather unstable, including all kinds of acquaintances that one would not consider as friends or family, but know well enough to have a conversation with or put names to their faces (Dunbar, 2016).

In this paper, we briefly review studies concerning the connection between people’s social network size and its underlying neural mechanisms. Throughout studies we find two social network size measurements that are widely-used in different studies. Upon closer examination, however, we spot the subtle difference between them, and subsequently find evidence from the underlying brain mechanisms to corroborate our findings.

## Social Network Size Measurements

At the operational level, how to measure the size of individuals’ multi-layer social networks is of practical importance. Throughout existing literature, two major types of measurements of social network size are frequently used.

Social Network Index (SNI) contains 12 different roles of people playing in their social networks. For each role, respondents are supposed to identify whether they have the particular relationship in the first place, and then choose the number of people they see or talk to on a regular basis (i.e., at least once every 2 weeks). Thus, the social network size can be computed by summing the total number of people in the 12 roles (Cohen et al., 1997; Peng et al., 2018).

Social Network Questionnaire (SNQ) and Norbeck Social Support Questionnaire (NSSQ) are the other frequently used types of measurements of social network size. In effect, the two questionnaires share many similarities. In SNQ, respondents are required to write down the names of their frequent contacts, and then they should identify those whom they would seek advice or comfort for a major personal problem like serious accidents or death of loved ones (Stiller and Dunbar, 2007). NSSQ requests respondents to list the names of network members who provide them with personal support and then rate each of their network members on a Likert scale by answering nine questions, such as “How much does this person make you feel liked or loved?” (Norbeck et al., 1981; Hampton et al., 2016).

## Comparison of the Measurement Tools

Comparing the above-mentioned two types of measurements about social network size (SNI and SNQ) in the perspective of social network organization, it can be found that they both focus on the primary inner layer of individuals’ social networks. However, the two assessments have subtle differences in essence as they measure the disparate sub-layers of social network size.

To be specific, what has been assessed in SNI is similar to the size of one’s sympathy group within a social network. Faced with SNI questions like “How many people at work do you talk to at least once every 2 weeks?” Respondents are very likely to include acquaintances without such a strong emotional bond, such as seeking personal advice or help in times of severe distress. However, what has been tested through SNQ can be regarded as the size of the innermost layer—the support clique of one’s social network, since one would always turn to those people for material and emotional support (Stiller and Dunbar, 2007; Ramirez and Palacios, 2016). In brief, compared with SNI, SNQ would arouse such a stronger affective feeling of being supported and adored as to remind respondents of the people at the core of their social network.

## Mri Findings

Advances in MRI analytics now provide tools to study brain–behavior relationships at the level of circuits and networks. Throughout studies on the connection between individuals’ social network size and brain mechanisms, the seemingly disparate findings can be pulled together to corroborate the subtle difference among social network size measurements.

### Studies With SNI Measurement

Bickart et al. (2011) performed a quantitative morphometric analysis of T1 weighted MRI data from 58 participants. The linear regression analysis reveals that individuals’ social network size is positively correlated with their amygdala volume.

Jasper (2013) investigated the correlation between the amygdala activation and social network size in HIV patients. In the research, emotional pictures of angry and fearful faces were displayed to illicit robust amygdala responses during an fMRI task. The result shows that there is a significant correlation between right amygdala activation and individuals’ social network size.

Dziura and Thompson (2014) recorded the motion of sensors attached to the limbs and torso of an actor and turned them into point-light arrays to present to the participants in an fMRI scanning. It is shown that the posterior superior temporal sulcus (pSTS), the amygdala and the fusiform gyrus are significantly activated in the perception of biological motions. Further exploration of the relationship between these cortical areas and social networks demonstrates that the amygdala and the pSTS activation are closely correlated with social network size. In addition, Lewis et al. (2011) conducted intentionality and memory tasks using a series of five short stories to test subjects’ ability to correctly infer the mind states like the beliefs of the characters in the story. The result shows that the ventromedial prefrontal cortex (vmPFC) volume predicts understanding of others and social network size.

Bickart et al. (2012) used three seed regions—the lateral orbitofrontal cortex (lOFC), the vmPFC and the caudual anterior cingulate cortex (cACC) to identify voxels within the amygdala with the strongest connectivity, thus, three subregions within the amygdala were parceled, being ventrolateral, dorsal, medial amygdala, respectively. In addition, they used these three subregions as seeds to conduct a whole-brain exploration, and built a network sharing functional connectivity with each amygdala seed. The result demonstrates that the ventrolateral amygdala and the medial amygdala networks can predict social network size.

### Studies With SNQ or NSSQ Measurement

Powell et al. (2012) focused on the PFC region and further divided the region into sub-regions, as dorsal and orbital prefrontal regions. The path analysis indicates that the orbital PFC volume is the best predictor of social network size.

Kanai et al. (2012) examined the correlation between gray matter density and social network size. The right amygdala density stood itself out to be significantly correlated with individuals’ social network size.

Heide et al. (2014) directed the participants to view their friends’ pictures and unfamiliar faces during an fMRI task. The result shows a significant BOLD activation in bilateral amygdala. In addition, the following VBM result indicates a positive correlation between gray matter volume in bilateral amygdala and bilateral OFC and social network size.

Similarly, Preller et al. (2014) conducted a social gaze task in an fMRI scanning, and found out a significant positive correlation between social network size and the medial OFC activation in the healthy control group.

Apart from that, white matter connectivity among different brain regions could also predict social network size as demonstrated in Hampton’s research. The diffusion-weighted imaging (DWI) result showed that the amygdala-OFC and the amygdala-ATL (anterior temporal lobes) white matter microstructure as well as age factor accounted for 69% of the variability in social network size (Hampton et al., 2016).

A careful examination can elicit an explicit tendency of all research results—SNI measured social network size is largely correlated with the amygdala, while SNQ assessed social network size is closely related to both the amygdala and the OFC.

## Discussion

The above-mentioned studies show that individuals with larger social networks have more gray matters and better function in brain regions implicated in adaptive social behaviors. The main goal of this review is to test whether the measurements of social network size are equivalent through neuroimaging study results. Taken together, our findings showed that structures and functions of the amygdala, the orbitofrontal cortex (OFC), the pSTS, and the vmPFC could predict the size of one’s social network. However, the OFC region was more saliently correlated to one’s innermost layer of social network, which revealed the subtle difference between the two measurements.

The fact that individuals with larger social network size have larger amygdala volume provides plausible evidence to the social brain hypothesis that primates evolved under the pressure of increasingly complex social life. The larger amygdala enables us to perceive social cues, and allow us to devise complex strategies to cooperate or compete with others more efficiently.

It is widely accepted that the amygdala is important for the recognition and processing of negative and positive emotions (Baxter and Murray, 2002; Dennison et al., 2015). When the pleasurable social cues are identified, the activation of the amygdala promotes social affiliation behaviors, adjusts social aversion behavior and improves interpersonal relationship in a larger social network (Preller et al., 2014). In addition, individuals with stronger intrinsic amygdala connectivity within other networks, for instance the perception network is better at decoding the meaning of social cues and dealing with larger amount of people in more complex social contexts (Bickart et al., 2012). To sum up, the amygdala enlargement or its activation or its structural and functional connectivity could predict the inner layer (both sympathy group and the support clique) of individuals’ social network size, regardless of the measurements being used. In contrast, another brain region—the OFC correlated with social network size is mostly identified by SNQ.

The OFC has been proved to be involved in a range of social functions. Intentionality is the ability to explain and predict the behavior of others. This social cognitive capacity has been demonstrated to be connected to the volume of the OFC (Powell et al., 2012). The result shows that a greater volume of the OFC means a better understanding of others, which contributes to maintaining a larger size of social network. Apart from that, previous studies found that the OFC volume or thickness could predict olfactory sensitivity (Frasnelli et al., 2010; Seubert et al., 2013), which in turn positively correlates to social network size (Zou et al., 2016). Even though the causal relationship is not clear, this result suggests that individuals with higher olfactory sensitivity are more sensitive to others’ body odor and can obtain more social chemical signals which facilitate social communication. Furthermore, empathy is the critical social skill in understanding what another person is experiencing (Preller et al., 2014); and the OFC activations were observed in empathetic behaviors (Matsudaira et al., 2017), which were more frequent among close or loved ones than unfamiliar companions (Romero et al., 2010). Those findings imply the connection, even though not causal relationship between the OFC activation and the innermost layer of one’s social network.

In addition, the anatomical location of the OFC is in the front end of the mesolimbic reward circuit (Rushworth et al., 2011), and it receives signals directly from visual, olfactory, taste, and somatosensory areas (Tanaka et al., 2016). Neuroimaging studies also found that the OFC was activated by pleasant touch, rewarding and aversive taste, and damage to the OFC impaired the learning of stimulus-reinforcement associations (Rolls, 2000; Dixon et al., 2017). As for the different types of rewards, social rewards like improving feelings of self-worth and importance through praise and the attention from others are the extremely important motivators for social interaction (Elliot et al., 2006; Izuma et al., 2008). Besides, recent studies showed that increased social interaction would enhance social reward, represented by the activation in the OFC, the mPFC, and the striatum of the reward system (Fareri et al., 2015; Kawamichi et al., 2016). Therefore, when assessed with SNQ which measures the most frequently interacted social network, the OFC activation would be more salient than that measured by SNI.

Apart from the ROI regions like the amygdala and the OFC, other brain regions are also found significantly related to one’s social network size, like the pSTS, the vmPFC, etc. It is widely acknowledged that the cognitive capacity of inferring the mental states of others is crucial to human sociality, according to the theory of mind (ToM; Frith and Frith, 2003). Neuroimaging studies have associated this ability with specific brain regions, as above-mentioned the pSTS and the vmPFC (Frith and Frith, 2003; Gallagher and Frith, 2003; Saxe and Kanwisher, 2003; Mahy et al., 2014). During ToM processing, the vmPFC is frequently active in identification of goals and intentions in a wide range of tasks (Gallagher et al., 2000; German et al., 2004; Lewis et al., 2011). In addition, the vmPFC is also proved to be involved in decoupling the perspectives of other people from one’s own (Gallagher and Frith, 2003). Equipped with theses capacities, individuals can infer the other person’s intention, separate out various layers between their acquaintances and themselves and maintain a large and stable social network. Turning to the pSTS, the recent study shows that this brain region responds strongly when perceiving social interactions (Isik et al., 2017). Besides, it is believed that the pSTS is involved in the perception of non-verbal social signals (Kanai et al., 2012; Dziura and Thompson, 2014), which can help reduce ambiguity and uncertainty. Therefore, greater functioning of the pSTS permits individuals to detect social cues and keep a larger size of social network (Goldin-Meadow and Beilock, 2010).

## Future Directions

Human’s social networks are hierarchically organized with different layers, which represent emotional bonds and interaction frequency among individuals. In terms of the measurement of social network size, SNI and SNQ are most frequently used. A careful examination of these two measurements in view of the hierarchical organization of social networks reveals that the two assessments are dissimilar in effect. Neuroimaging researches shed light on a new perspective as they uncover the underlying neural mechanisms of human’s social networks. Throughout the existing literature, social network size measured by SNI is largely correlated with the amygdala, while social network size assessed by SNQ is closely related to both the amygdala and the OFC, which provides evidence to the subtle difference between the two measure tools. This finding sheds new light on the understanding of the subtle distinctions among various social network assessments and suggests that we should choose the most suitable one for specific research purpose, since our brain would react distinctively to social interactions with dissimilar emotional closeness.

In recent years, the rise of the Internet has provided an opportunity to study social networks on a larger scale (Hayat et al., 2017). A key element of social networks is the ability for individuals to simultaneously interact in multiple social contexts by maintaining different types of social ties. The overlay of several networks on the same set of nodes (individuals) is called a multiplex network (MPN). The MPN facilitates the description, quantification, and analysis of complex sets of relationships among individuals (Hayat et al., 2017; Bilecen et al., 2018). Lately, Parkinson et al. (2018) proposed a new approach to characterize individuals’ social network. They recruited an entire cohort of students in a graduate program and asked them to complete an online survey in which they indicated the individuals in the program with whom they were friends. Given that a mutually reported tie is a stronger indicator of the presence of a friendship than an unreciprocated tie, a graph consisting only of reciprocal social ties was used to estimate social distances between individuals.

Future studies could also focus on other dimensions of social network like its diversity and embeddedness. In SNI, social network diversity is represented by the number of social roles in which the participants have regular contact with at least one person; and social network embeddedness is represented by the number of different network domains in which a participant is active (Dziura and Thompson, 2014; Molesworth et al., 2015). Which layer do these measurements exactly focus on? Do they assess the same thing in essence? Answers to these questions would provide us with better comprehension of the essence of measurements and a new perspective of balancing the advantages of each measurement against their shortcomings.

## Author Contributions

XZ, XL, and SL conceived and designed the writing frame. XL and SL wrote the paper. XL, SL, RH, XC, YX, RM, YL, JB, and XZ revised the manuscript.

## Conflict of Interest Statement

The authors declare that the research was conducted in the absence of any commercial or financial relationships that could be construed as a potential conflict of interest.
